# The Extraction of the First Permanent Molar

**Published:** 1891-07

**Authors:** Eugene J. Wetzel

**Affiliations:** Mulhouse, Germany


### THE EXTRACTION OF THE FIRST PERMANENT MOLAR.[1]

BY DR. EUGENE J. WETZEL, MULHOUSE, GERMANY.

The usual cause of premature extraction is caries, and this has also to be resorted to in cases of overcrowded dental arches; no blame can therefore be attached to the operator who has acted with proper judgment.

Our present style of living differs greatly from that of the past; the climate is undergoing gradual changes, and habits also change with the advance of civilization. The food now taken does not require the same mastication as that formerly used; the nervous system is more irritable, due to high pressure, education, and social conditions. The effect of these changes is, that while the jaws are growing smaller the dermal appendix remains the same, and we see overcrowded arches, irregularities, and caries more frequently.

The first teeth of the permanent denture which are erupted are the first molars, this occurring at the sixth year.

Physiologically, the first molar is the most useful tooth in the whole arch; it presents the largest area of crown-surface, is situated in a position where mastication is greatest, and is admirably adapted to bear strain.

It is more liable to caries than any other tooth, caused probably by imperfect structure, on account of its development at the period when nutrition in the child is often at fault.

In the sixth-year molar, then, we have two great characteristics, its utility in mastication, and its tendency to decay. These teeth often show some signs of caries when their grinding surface is hardly free from the gum, and a few months after there are very large cavities to be seen. I think I can safely say that no more than six per cent. of these teeth are spotless and of good quality, which is a sadly small number.

These teeth, then, have to be watched from a very early period and filled, and if not of a medium quality have always to be repaired.

Since the first molars are so liable to decay, it will be found at all times a difficult matter to put a permanent filling in these teeth.

---

[1] Read at the meeting of the Harvard Odontological Society, by Dr A. H. Stoddard, Boston, November 29, 1890.

When considering our treatment we should bear this fact in mind, should such a filling fail we are worse off than ever. I should therefore advise the extraction of all first molars if premature decay has attacked them, and we then have no certainty of introducing a permanent filling.

Having in an overcrowded mouth moderately sound first molars, there arises the question, Should some one of the bicuspids or the molars be removed?

For this a very fixed rule cannot be stated; but I think to remove teeth in order to save others from caries is not at all bad practice. We may safely say that if a first molar remains good until the age of twelve, and has withstood the secretions of the mouth, it may safely be kept in place.

There is no doubt that there are cases in which we should adopt radical treatment, and if only two such teeth are carious I should strenuously advise the removal of all four. The spaces left will be gradually filled by the twelfth-year molars and the second bicuspids. Should one sixth-year molar be extracted, the opposing tooth will be locked (and the space remains). It is then useless for mastication, and caries will make rapid progress.

Presuming extraction has been chosen as treatment, the question arises, When should it be done?

Should we extract the first permanent molars before our little patient has attained the age of twelve? Should they be carious we should employ all our means to fill them in order that they may last until the age specified for extraction. I think the best time for their removal is when the twelfth-year molars have fully erupted, and their grinding surfaces touch each other; their forward movement is then more even.

Should all the four teeth be removed at one sitting? I think not; a better way is to remove first on one side, waiting till the wound is pretty well healed and the patient is able to masticate on that side again. There is no harm to wait two, four, six, or eight months. If they are removed all in one sitting, the patient will have to wait several weeks till able to masticate on the sides again, and the neighboring teeth will suffer considerably.

The best filling for such teeth is Sullivan's amalgam (or what is called in the States copper amalgam), it has the tendency to harden softened dentine.

